# L’ansa pancreatica: une cause rare de pancréatite aigue

**Published:** 2012-10-16

**Authors:** Hichem Ayari, Saber Rebii, Manel Ayari, Radhouane Hasni, Ayoub Zoghlami

**Affiliations:** 1 Service de chirurgie générale Centre de Traumatologie et des Grands Brulés, Ben Arous, Tunisie

**Keywords:** Ansa pancreatique, pancréatite, voies biliaires, Ansa pancreatique, pancreatistis, Bile duct

## Abstract

L’ansa pancréatica est une voie de communication accessoire entre le canal de Wirsung et un conduit pancréatique accessoire ne présentant pas de jonction normale avec le premier. L’association entre cette variante anatomique et la pancréatite aigue dite idiopathique reste hypothétique. Nous rapportons l’observation d’un patient présentant des poussées de pancréatites récidivantes qui serait en rapport avec une Ansa pancréatica.

## Introduction

L’ansa pancréatica est une voie de communication accessoire entre le canal de Wirsung et un conduit pancréatique accessoire ne présentant pas de jonction normale avec le premier. L’association entre cette variante anatomique et la pancréatite aigue dite idiopathique reste hypothétique. Nous rapportons l’observation d’un patient présentant des poussées de pancréatites récidivantes qui serait en rapport avec une Ansa pancréatica.

## Patient et observation

**Figure 1:**
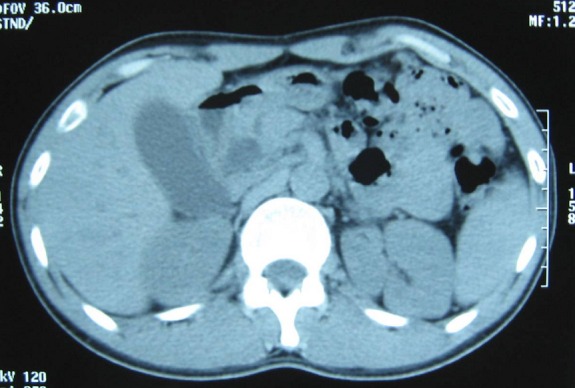
TDM abdominale sans injection de produit de contraste

Monsieur AB âgé de 28 ans admis en décembre 2006 pour des douleurs épigastriques d’apparition brutales, transfixiantes et sans fièvre associée. L’examen physique est normal. A la biologie, il existe une hyperamylasémie à 6 fois la normale. L’échographie abdominale faite en urgence a montré une vésicule biliaire distendue, à paroi épaissie mesurée à 5 mm et alithiasique. La voie biliaire principale était dilatée à 10 mm sans dilatation des voies biliaires intra-hépatiques. Le scanner abdominal fait à J 3 d’évolution a montré une pancréatite aigue stade C. Les suites étaient favorables avec une disparition des douleurs épigastriques et une restitution ad integrum des lésions sur le scanner abdominal de contrôle. L’enquête étiologique comprenant un bilan lipidique et phosphocalcique était négative. Par ailleurs il n y avait pas de notion d’éthylisme chronique. Ensuite, le patient a présenté une poussée de pancréatite aigue par an. La dernière poussée remonte au mois de Décembre 2010. Une échographie abdominale a montré une vésicule biliaire à paroi fine et à contenu transonore. La voie biliaire principale faisait 13 mm, Les voies biliaires intra-hépatiques n’étaient pas dilatées et le pancréas était de taille normale et d’échostructure homogène. Un scanner abdominal fait à J3 d’évolution a conclu à une pancréatite stade B (Figure 1).

**Figure 2:**
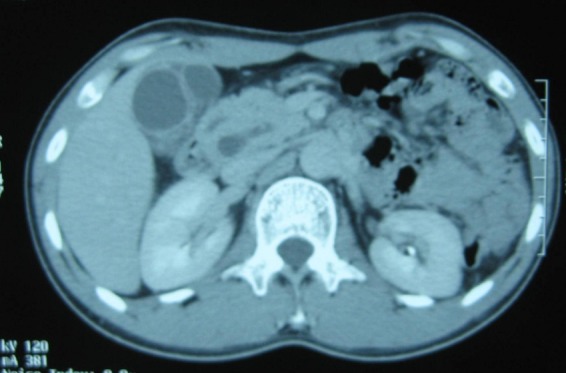
TDM abdominale après injection de produit de contraste

Devant cette pancréatite récidivante une wirsungo-bili-IRM a été faite et avait montré une dilatation des voies biliaires intra et extra-hépatiques avec une image du bas cholédoque, interprétée comme étant un petit calcul, une dilatation du canal de Wirsung et la présence d’un canal accessoire type ansa pancréatica (Figure 2).

**Figure 3:**
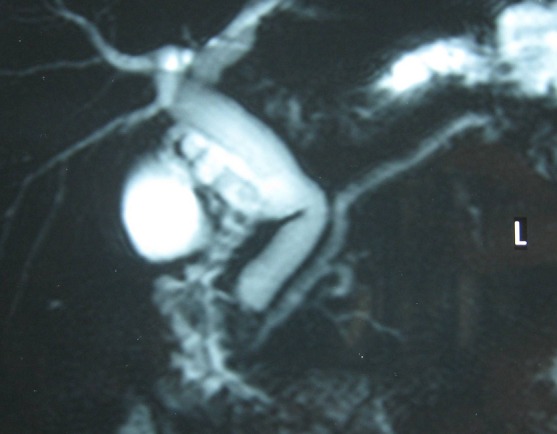
Wirsungo-bili-IRM: un canal pancréatique accessoire reliant les deux canaux principal et accessoire

Une sphinctérotomie endoscopique a été tentée pour assurer la vacuité de la voie biliaire principale. Celle ci a montré une papille accessoire augmentée de taille et une papille principale fine et non cathéterisable. Il a été décidé ainsi d’opérer le patient. En per opératoire; la vésicule était distendue et la voie biliaire principale était dilatée. Après la cholécystectomie, la cholangiographie trans-cystique (Figure 3) a montré un moignon cystique, une voie biliaire principale et un canal de Wirsung qui sont dilatés, et surtout elle a révélé la présence d’un canal accessoire type ansa pancréatica.

**Figure 4:**
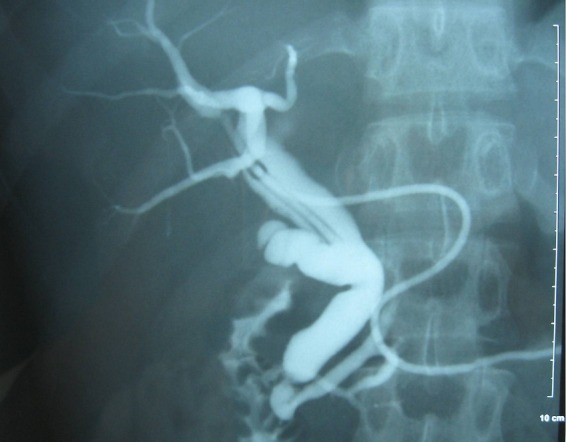
Cholangioragphie per opératoire: aspect en faveur d’une ansa pancréatica

La cholédocotomie sus duodénale a permis de vérifier la vacuité de la voie biliaire principale et en particulier l’absence de calcul à son niveau. Le drainage des voies biliaires a été assuré par un drain de Kher. Les suites opératoires étaient simples et la cholangiographie postopératoire a montré des voies biliaires intra et extra-hépatiques libres et un canal accessoire type ansa pancréatica qui draine le Wirsung par la papille accessoire (Figure 4).

## Discussion

L’ansa pancréatica est une variante décrite pour la première fois par Dawson en 1961. Il s’agit d’une voie de communication accessoire entre le canal de Wirsung et un conduit pancréatique accessoire ne présentant pas de jonction normale avec le premier. Elle serait formée par la jonction des branches inférieures des canaux pancréatiques principal et accessoire. Dans cette configuration, la papille mineure semble être le plus souvent perméable.

**Figure 5:**
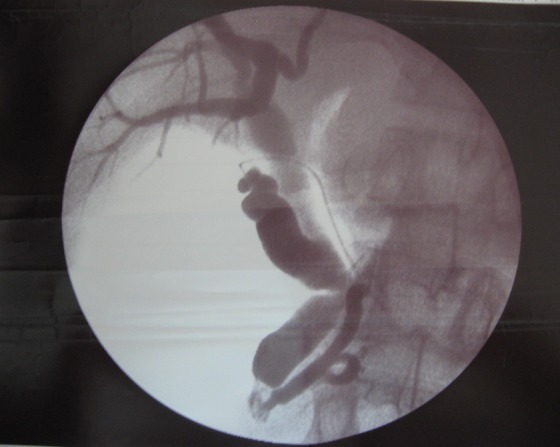
Cholangioragphie per opératoire: aspect en faveur d’une ansa pancréatica

Dawson et Langman ont appelé ansa pancréatica la formation dans laquelle une communication entre les branches inférieures des canaux de Wirsung et Santorini supplée à l’absence de jonction normale de ceux-ci; cela induit une image en boucle bien reconnaissable en imagerie [1] et qu’on arrive à identifier sur les différents clichés d’IRM ou d’opacification des voies biliaires réalisés chez notre malade [1] (Figure 5).

Simkins [2] a émis l’hypothèse que ces jonctions aberrantes soient le résultat d’un développement initial plexuel des canaux pancréatiques durant l’embryogenèse, seuls persistant au final ceux dans lesquels un flux suffisant s’écoule; cependant, aucune étude embryologique n’est venue étayer cette hypothèse [2]. Kamisawa reprend quant à lui cette "théorie des flux" pour décrire des canaux de Santorini courts ou longs dont la formation serait dépendante du flux antérograde ou rétrograde dans le canal de Santorini [3, 4].

La relation entre l’ansa pancréatica et la survenue d’une pancréatite aigue reste hypothétique et controversée [5]. Elle serait expliquée par l’abouchement de l’ansa pancréatica dans le canal de Wirsung selon un angle oblique entravant ainsi le bon drainage de la glande pancréatique et provoquant par la même occasion des pancréatites aigues selon le même mécanisme dans les sténoses du sphincter d’Oddi et pour les origines alcooliques [6–8]. Une pancréatite aigue peut survenir chez 3- 31% des patients ayant une variante anatomique des canaux pancréatiques [9]. D’après Hiroshi [10] environ 7% des patients ayant une ansa pancréatica présentent une pancréatite aigue. Selon ce même auteur, cette variante anatomique peut être découverte lors des explorations faites pour une autre pathologie à savoir une dilatation kystique du cholédoque (87%) ou une lithiase biliaire (7%) [10]. Actuellement, l’IRM est entrain de révolutionner l’étude des canaux biliopancréatiques en devenant l’examen diagnostique de première intention [11]. Elle est indispensable pour le bilan étiologique des pancréatites aigues non biliaire récidivantes [12].

## Conclusion

L’ansa pancréatica peut être considérée comme un facteur prédisposant aux pancréatites aigues idiopathiques. La pancréatographie par IRM permet de reconnaitre cette variante anatomique en préopératoire quand elle est symptomatique.
